# Ten simple rules for using entrepreneurship skills to improve research careers and culture

**DOI:** 10.1371/journal.pcbi.1009946

**Published:** 2022-04-08

**Authors:** Matt Bawn, David Dent, Philip E. Bourne

**Affiliations:** 1 Earlham Institute, Norwich Research Park, Norwich, United Kingdom; 2 Quadram Institute, Norwich Research Park, Norwich, United Kingdom; 3 University of East Anglia, Norwich Research Park, Norwich, United Kingdom; 4 University of Virginia, Charlottesville, Virginia, United States of America

## Introduction

The academic path is not an easy one. The acknowledgment of the problems of the research precariat [1], the lack of stability and certainty in our careers, is not a new thing. The number of permanent positions in academic research has always limited the career prospects of many postdocs, but there are concerns the increasing supply of graduates is exacerbating this issue [2], and for scientists, job satisfaction is at an all-time low [3]. Indeed, the current situation is forcing many of us to reevaluate our options [4]. For many, this may mean leaving the academic world and moving to industry or enterprise, but this often can feel like failure [5]. We argue that the establishment of a scientific career can be thought of as an entrepreneurial enterprise. We contend that an acknowledgment of this viewpoint and reevaluation of what constitutes value and impact in this system can contribute towards an improved research culture. With this in mind, we present some rules informed by the world of enterprise and business to help the **early career researcher** develop and navigate their careers.

Entrepreneurship can be defined as the means by which new organisations are formed with their resultant job and wealth creation [6]. We can refocus this definition on the individual and define career entrepreneurship as the process of developing a career that results in increased value for the individual. Some common traits have been identified in entrepreneurship: risk taking, achievement, autonomy, self-efficacy (the ability to complete tasks), and locus of control (the control we believe we have on the outcomes of our lives [7]) [8], and these traits are also important markers and indicators for career progression in scientific research.

Druker describes innovation as the ability to exploit change and argues that looking for change and exploiting it as an opportunity defines an entrepreneur [9]. Change is a commodity that is not in short supply in the world of scientific research, with changing political and funding landscapes as well as new discoveries and knowledge. Through the definitions and perspectives offered above, we can prepare ourselves to be able to navigate and exploit changes for the benefit of our careers using traits and techniques also found in entrepreneurship and in innovators.

### Rule 1: Be wary of rules

It may be surprising that this is the first rule of a 10 simple rules article, but it is important to acknowledge that in something as important as career progression and research culture, there are no hard and fast rules. Indeed, in some situations too many rules can be the enemy of innovation [10]. You could even make the argument that a willingness to challenge rules is an entrepreneurial trait [11]. However, not everybody is in the same situation, and for rules like Rule 10: Don’t be afraid to take risks, personal circumstances may dictate other courses. The purpose of this article is, therefore, to offer the reader some food for thought, a series of viewpoints gained through our experiences that the reader may not previously have considered that may help them look at their personal situation from a different point of view and which, in turn, may provide some use. Opposing advice may exist for many of the following rules [12], and it is up to the reader to decide the value with respect to their own personal circumstances.

### Rule 2: Recognise and utilise networks

Social and professional networks run through all aspects of our lives and describe who we know, who we associate and work with, as well as our friends and family. When we as scientists think of networks and networking, we tend to think of conference wine receptions, but we need to remember that many different networks run through our daily research lives: from the administrators and support staff that help manage the research to professors and directors that lead it. If you can identify one person at a conference or expert or agency that can help progress you and your work, then think about how to begin a successful interaction. Science can often be much more hierarchical than business—one consequence of this is that we are often pigeonholed based on age and career stage, which can make independently establishing relationships challenging. To do so requires some time and preparation. Why would that person want to talk to you? What value could your conversation bring them? Do you have any mutual contacts that could facilitate a conversation? For these things to fall into place, you also need to clearly know what it is that you want. Do you want them to be on a grant with you, support a fellowship application, or just listen to an idea? In each case, think why they would want to spend their time helping you and try and show them why doing so would benefit them. If you know that they will be at a conference that you will also be attending, email them beforehand and suggest meeting up. Although this may be hard the first time, the next time, after some level of success, it will be easier, and you will be better prepared.

### Rule 3: Create a personal narrative

The entrepreneur Marc Andreessen said that “A company without a story is a company without a strategy,” but we could equally say, a researcher without a story is a researcher without a goal. What vision do you have, and hence what choices have you made in your research career? How have you developed your interests and shaped your career trajectory? What personal obstacles have you had to overcome, and how have they shaped who you are today? These are questions that are asked of all of us at every stage of our development, and it’s good to reflect on how you have got to where you are and what vision drives you to where you want to go and construct this as a story and practice telling it. The word trajectory is important here; it implies not only a career goal but also that you are moving along a path to reach it as part of an overarching vision. In other words, you’re going places, and it’s that dynamism that you need to bring into your narrative.

A good personal narrative that expresses your vision helps others see you how you want to be seen and explains how any of your experiences have value, not necessarily just those directly related to your research. This is often not an easy thing to achieve. Too often as scientists we strive for the impersonal. In our quest to be as objective, independent, and scientific as possible, we remove the human element from the narrative, but we need to remember people are at the heart of all our research stories, and our success and progression ultimately depends on the relationships (**see below**) we forge through how we project ourselves.

### Rule 4: Broaden your networks

Perspective is an important element of personal development. To move forward, it is a good idea to know where you are, where you want to go, and also have an idea of how to get there (Rules 3 and 5) but also who could help or hinder us along the way. This is not always as easy for scientists concentrating on very specific research questions. During PhD studies, we spend years focusing on our research topics entrenched in our research groups. As postdoctoral researchers, it’s easy to fall into the same pattern and become myopic in our focus. It’s a common academic mantra that career progression depends on publications and so we sacrifice activities that don’t relate directly to our research and manuscripts. Publications are undoubtedly important, but there will always be someone who has more publications than you, so we must also strive for balance. Building relationships with people outside of the research environment, experiencing different cultures and approaches, and learning in a nonacademic context are important for personal development and will help us orient ourselves and understand the rules and languages in unfamiliar research institutions and cultures [13]. It is an essential skill in all areas of life and one that is often overlooked in research environments, but larger networks and greater interaction with peers and institutions often lets us see the “bigger picture” that we need to ground our research and find the next idea and opportunity.

### Rule 5: Reflect on goals and strategies

It could be said that in a research landscape, goals are destinations and strategies are the routes we take to get to them. For many of us, one of the driving forces in career progression is the desire to be independent researchers. We imagine that independence means the ability to choose where we want to go and how to get there.

Where do you want to go and what’s stopping you?

“Reflection is particularly important for perplexing experiences, working under conditions of high uncertainty, and problem-solving. As a result, it should not be a surprise that reflection is an integral component of entrepreneurship education and also a way of practicing entrepreneurship” [14].

Where are we on our research trajectory (Rule 3)? Are we where we are because of or in spite of our surroundings (Rule 4)? Can we do anything to improve our situation (Rule 2)? This process of reflection helps us locate ourselves within our narratives to more easily identify our goals and plans to achieve them. However, “no plan is an island,” and goals have hierarchies and are frequently fickle and subject to change, and so we must be flexible. The solution might not be what was anticipated, but this does not invalidate having a goal or a strategy to achieve it. How do our goals fit within research themes, institute, or even research funder strategies? By addressing the previous steps through self-reflection, we can attempt to answer this question. Projects that obviously fit into stakeholder and environmental strategies stand more chance of being funded or championed, but we need to ensure that the strategies truly “align”; this means not simply using the same keywords or zeitgeist buzzwords but identifying tangible links that lend purpose and direction to your research and orient it within the larger research environment. This also helps us learn to say no to “opportunities” that don’t help us reach our goals, which is a difficult but necessary step towards becoming more effective researchers.

### Rule 6: Develop self-management skills

Management at its most basic means utilising all the resources available to you effectively in order to achieve your goals. As early career researchers our access to resources is limited, although perhaps paradoxically, a demonstration of how we have won personal research funding or managed research is often a requirement for career progression. It’s therefore up to the entrepreneurial researcher to seek these out. Some, like early career grants for travel or research, are relatively easy to find but are often highly competitive. But there are lots of potential opportunities, writing a proposal for a project student could lead to personal and project management experience as well as preliminary data that could be used for a larger application. Perhaps the most important but limited resource that all researchers must learn to manage is time. At all stages of our research careers, we are faced with multiple tasks and deadlines, and many of these different tasks could be “urgent” so it’s important to know where to focus our attention to not be overwhelmed but efficient and effective. There are many time management techniques and tools available [15,16], but how effective they are may vary from person to person, so try them out and see what works for you. In our personal experience, we find it useful to break tasks down so that we can always achieve something each day. We try and maintain motivation by feeding it a succession of these little successes that hopefully will lead to the big goals we ultimately want to achieve.

### Rule 7: Appreciate the language needed for interdisciplinary interaction

Innovation spans disciplines increasingly so does research. Hence, while it is comfortable to imagine one academic language, we need to be aware there isn’t and to take care that we really try to communicate effectively. Just as when learning a foreign language, we shouldn’t assume that we will understand exactly what is being said without making the effort to learn some key words and phrases. A major pitfall we need to be aware of is the frequent use of terms that have different meanings in different disciplines: ontological faux amis. These trick us into thinking we understand what is being said when in fact we may be just filling in the blanks. This is a natural linguistic response that our brain automatically does in everyday communication; it uses past experience to make a “best guess.” However, we need to be conscious that this happens in our science conversations too, sometimes perhaps intentionally, and that the longer we work in a field, the more fixed become the experiences from which we backfill. We must look at the multidisciplinary team doing the science in the same way we look at a scientific instrument. If you don’t put the effort in at the beginning learning how to use it properly and what all the buttons and settings mean, then how can you have any confidence in the results? This also means we need to get over our embarrassment about asking questions for as Confucius reportedly said, “The man who asks a question is a fool for a minute, the man who does not ask is a fool for life.”

### Rule 8: Recognise interaction as being complex

We need to recognise that how we interact with each other depends on lots of different factors [17]. Recognising that there are frameworks for learning [18] and techniques [19] that have been developed that can be applied in directing, and integrating research from different scientists to achieve a common goal is a useful step. Actually applying those techniques can transform the outputs and delivery of multidisciplinary research programmes. Formal training for scientists helps raise the importance of the issues and means by which problems can be avoided and solutions delivered using multiple skill sets. Integration of research outputs can occur at the end of a programme by simply preparing a report where editorial devices are used to combine and link different elements of the programme. However, most effective integration is possible if organised from the outset using common group learning [20], negotiation among experts, modelling, or integration through leadership—different sociocognitive frameworks of integration. There is a large body of work relevant to this with respect to synthesis centres—goal oriented, multidisciplinary research centres, specifically implemented to catalyse collaboration and lead to break through ideas [21]. Indeed, in a manner of speaking, the rules presented here aim to make the reader into synthesis centres capable of achieving breakthroughs in their career development.

### Rule 9: Recognise markets and communicate accordingly

Why do we do research? Who is the intended “consumer” who will read our articles? Who is interested, and who will the findings affect? Trying to answer these questions can help us increase the impact and value of what we do. Most research is scientific supply led rather than market or stakeholder led. How is research going to be directly delivered to stakeholders? Current science is delivered as a modular process providing neat packages for potentially interested parties and those not involved in the research. Scientists need to take ownership of delivery of their work to stakeholders. This means taking ownership of explaining the findings directly to the public or other audiences via blogs or talks and, for scientists in academia, also developing links with industry to lead the knowledge exchange process (aka translation). There is already a move towards more effective science communication, but often this is seen as a secondary output of the research, something for the postdocs or PhD students to do and not as important as the paper. Undoubtedly, the paper is vital, but the effort to disseminate the research findings still needs to be maintained past its acceptance. The growing call to demonstrate the impact of our work beyond the academic sphere can help us realise that the academic presentation and paper, the blog, press release, OpEds [22], and discussions with industry or policy makers are all part of delivering science. Ultimately, by attempting to communicate our work in different ways and with different groups and listening to how that research is received, we can get a better idea how it and, therefore, we as researchers, fit into societal demands.

### Rule 10: Don’t be afraid to take risks

One of the key characteristics of an entrepreneur is that they are prepared to take risks—often risking everything in the pursuit of an idea. Early career scientists take risks as part and parcel of their training and research roles. They need to be open to moving to new locations and institutions and flexible in the roles available and always careful and not to become too specialised too early all add elements of risk. However, it is a habit worth forming: taking risks and “putting yourself out there,” being prepared to do things differently, creating and seizing opportunities and not being afraid of doing so. There is, at the present time, a sort of academic production line; people go in at the beginning of their university careers by studying as an undergraduate, then a PhD, a couple of postdoc positions, and it’s time to start looking for more permanent positions. How can we stand out along the way?

We need to be prepared to ask for forgiveness rather than ask for permission when working with the uncertainties of a research career. Make the risks work for you—in developing a portfolio of capability—building your narrative of where you want to go and then—go there!

## Conclusions

The behaviours, values, expectations, attitudes, and norms of our research communities are what constitutes research culture, and this is what determines how research is carried out and shared and shapes the way that researchers careers develop. Research culture can exist on many different scales: from research group to institution to national or even international levels.

In the United Kingdom, the recognition of the changing landscape in which science is being delivered has led to UK Research and Innovation (UKRI) and government working together to shape policy to try and create a more innovative, impactful, and sustainable research environment through the research and development roadmap [23] and through the concordat to support the career development of researchers.

The recognition that there can be more than one career path and an acceptance that researchers can and sometimes should freely move between academia and industry to help their careers will hopefully lead to stronger and more innovative research. We hope that by thinking differently about the way we do things, by taking a leaf out of the entrepreneurs’ books, and by following these 10 simple rules, we can make these transitions a little easier and smooth the way for more equitable routes to impact a more sustainable research ecosystem [8]. We offer some general advice that will help the researcher navigate their careers or at least help them think about it from a different perspective to increase their survivability [24] and promote the retention of a diverse cohort of scientists [25] to contribute towards a better research culture.
